# A New Proposal for Severity Evaluation of Menière's Disease by Using the Evidence From a Comprehensive Battery of Auditory and Vestibular Tests

**DOI:** 10.3389/fneur.2020.00785

**Published:** 2020-08-18

**Authors:** Shujian Huang, Huiqun Zhou, Enhui Zhou, Jiajia Zhang, Yanmei Feng, Dongzhen Yu, Haibo Shi, Jian Wang, Hui Wang, Shankai Yin

**Affiliations:** ^1^Department of Otorhinolaryngology-Head and Neck Surgery, Shanghai Jiaotong University Affiliated Sixth People's Hospital, Shanghai, China; ^2^Otolaryngology Institute of Shanghai Jiao Tong University, Shanghai, China; ^3^Shanghai Key Laboratory of Sleep Disordered Breathing, Shanghai, China; ^4^Department of Otolaryngology-Head and Neck Surgery, Shanghai Gongli Hospital, The Second Military Medical University, Shanghai, China; ^5^School of Communication Science and Disorders, Dalhousie University, Halifax, NS, Canada

**Keywords:** Menière's disease, dizziness handicap inventory, vestibular-evoked myogenic potential, rotatory chair test, video head impulse test

## Abstract

To date, no widely accepted criteria exist to quantify the severity of Menière's disease (MD) by using vestibular tests. This study aimed to compare association of hearing loss and vertigo severity with association of accurate assessments of vestibular function and the vertigo severity. The severity of vertigo was documented by a comprehensive medical history with number of vertigo attacks in the past 6 months and a Dizziness Handicap Inventory (DHI) questionnaire. The involvement of vestibular organs was verified by audio-vestibular tests in 80 definite MD patients. Correlations between DHI scores, number of vertigo attacks in the past 6 months, audio-vestibular function, and the number of involved vestibular end organs were evaluated. We show that there are no significant differences in either severity of vertigo or laboratory results across the degree of hearing loss. Furthermore, the number of involved vestibular end organs was significantly correlated with vestibulo-ocular reflex gain in anterior and posterior canal video head impulse test (vHIT), interaural asymmetry ratio in vestibular-evoked myogenic potentials (VEMPs), and number of vertigo attacks in the past 6 months and DHI score. The vestibulo-ocular reflex gain in the rotatory chair test (RCT) was significantly correlated with the DHI Physical scores and number of involved vestibular end organs at 0.08 Hz. These results indicate that hearing loss is a poor indicator of vertigo severity in MD whereas the number of involved vestibular end organs may serve as an objective measure for MD progress. A battery of vestibular tests targeting different sensor organs is a complementary method for evaluating inner ear deficits and may aid in “grading” the severity of MD.

## Introduction

Menière's disease (MD), named after Prosper Menière, is an inner ear disorder characterized by spontaneous episodes of vertigo attacks (1), which is often accompanied by fluctuating sensorineural hearing loss (SNHL), tinnitus, and aural fullness (2–4). The prevalence of MD has been estimated to be 0.27% in the United Kingdom on the basis of cross-sectional data over 500,000 participants in the UK Biobank collected between 2006 and 2010 (5). A study in the United States reported an estimated prevalence of 0.19% on the basis of information obtained from a health claims database between 2005 and 2007, which included 60 million records (6). MD is a complex, heterogeneous disorder in which numerous underlying factors interact, including anatomical variations in the temporal bone, autoimmunity, and altered intralabyrinthine fluid dynamics due to abnormal functions of ion channels and transporters (1, 7–9). Endolymphatic hydrops (EH) due to overproduction of endolymph is a common pathology in MD, as it has been verified in anatomical studies of temporal bones (10–12) and more recently in MRI studies (13–16). However, the exact etiology of EH and its role in MD are still poorly understood (8, 9, 17).

Peripheral episodic vertigo attacks are the major symptom and disabling cause in MD and are likely brought up by a malfunction in vestibular system organs rather than representing a cochlear pathology (7). However, the severity of MD has been quantified mainly based on the degree of SNHL. As proposed by the Equilibrium Committee of the American Academy of Otolaryngology-Head and Neck Surgery (AAO-HNS) in 1995, MD is categorized in a four-stage system mainly based on pure-tone average (PTA) thresholds but not vestibular dysfunctions. More recently, the 2015 diagnostic guideline formulated by the Classification Committee of the Bárány Society and other international societies (17) emphasized the diagnostic significance of the combination of a history of episodic vertigo attacks and low- to middle-frequency SNHL. However, no revision was included in the new guideline on how the severity of MD should be quantified. Because the major symptom of MD mainly affects balance, not hearing, this staging system may not represent the natural severity development of this disease.

Although MD is mainly a balance disorder, currently, no vestibular test specific for the diagnosis of MD is available (18–20). Moreover, no widely accepted set of criteria exists to quantify the disorder using vestibular organ tests. In fact, laboratory tests to examine individually the functions of all vestibular organs are lacking, and to date, quantitative measurements of these functions are not available. The major symptom of MD, the vertigo attack, is episodic and temporarily self-restrained, likely owing to the adaptation in the entire vestibular system and compensation by other sensorial functions (21). The adaptation and potential compensation further increase the difficulty to evaluate the degree of pathology in the vestibular organs on the basis of the clinical symptoms.

For a long time, the vestibular test was mainly focused on the horizontal semicircular canal (H-SC) owing to technical difficulties and equipment constraints. With progress in the last decade (22–25), tests addressing the functions of individual vestibular organs have been developed and matured. It is now possible to establish a new quantification system that is based on functional disorders in the vestibular organs rather than solely on the low-frequency SNHL. Specifically, the rotatory chair test (RCT) examines the function of the H-SC and the superior vestibular nerve in the vestibulo-ocular reflex (VOR) initiated by low-frequency rotation; the video head impulse test (vHIT) targets the function of horizontal, posterior, and anterior SCs (H-, P-, and A-SC) in high-frequency VOR; the cervical and ocular vestibular-evoked myogenic potentials (cVEMPs and oVEMPs) target the functions of the saccule (inferior vestibular nerve) and utricle (superior vestibular nerve), respectively. Therefore, a test battery including these four tests can assess the involvement of all five vestibular organs.

In the present report, we performed correlation analyses between the results of the four vestibular tests and the clinical symptoms in patients affected by MD by employing a moderate sample size. Our primary aim was to evaluate a correlation between accurate assessment of hearing loss, vestibular function, and vertigo severity and to provide evaluation proposals for the inner ear deficits in patients with MD.

## Methods

**Figure 1:**
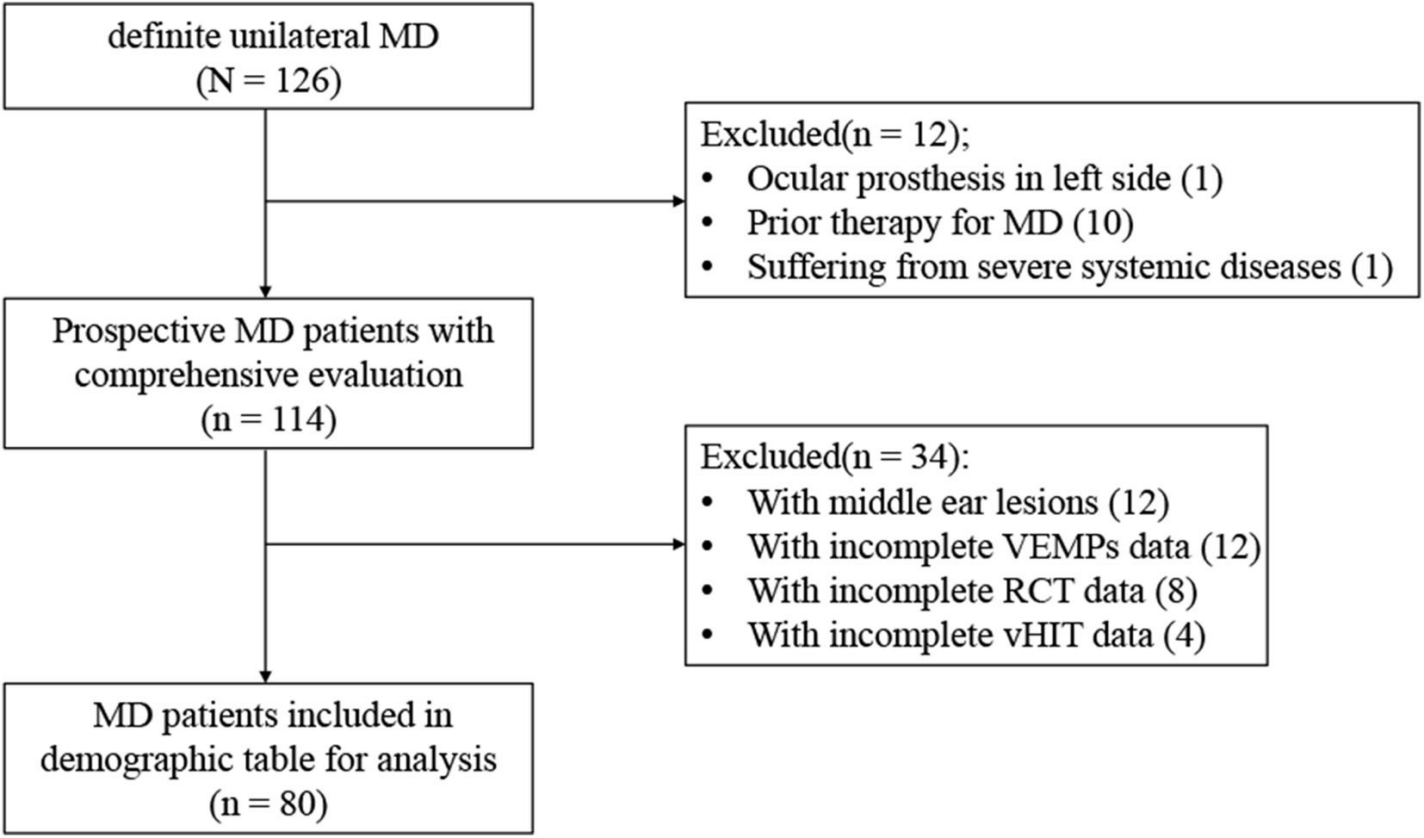
Selection process.

A total of 80 patients with MD were recruited between August 2018 and October 2019 from the ENT clinic of the Sixth People's Hospital affiliated to the Shanghai Jiao Tong University. A comprehensive medical history was taken to obtain demographic and clinical information. An otoscopic examination was carried out to rule out abnormalities of the external or middle ear. The inclusion criteria included (1) age between 18 and 85 years; (2) presented with two or more spontaneous episodes of vertigo lasting 20 min to 12 h, audiometrically documented low- to medium-frequency hearing loss on at least one occasion, and fluctuating aural symptoms such as tinnitus or aural fullness in the affected ear, excluding another vestibular diagnosis, which made up a diagnosis of definite unilateral MD according to the Bárány Society guideline of 2015 (17), (3) clear understanding of the purpose of the study (by the patient or his/her legal representative), and (4) the ability to comply with the research program. The exclusion criteria included (1) severe cardiovascular or cerebrovascular diseases or organ failure; (2) severe cervical or lumbar diseases, fractures, or craniocerebral trauma; (3) middle ear lesions, acoustic neuroma, ear trauma, barotrauma, large vestibular aqueduct syndrome, or other congenital cochlear malformations; (4) having participated in or undergoing a clinical trial of any drug or medical device within 3 months prior to the study that may impact auditory or vestibular functions; (5) having central vertigo, severe neurological, or psychiatric disorders; and (6) being a pregnant or lactating female. The exclusive selection process is summarized in Figure 1.

After the recruitment, every participant (or his/her legal representative) was asked at the first visit to complete the informed consent form and the Dizziness Handicap Inventory (DHI) questionnaire. This was followed by a comprehensive audiological evaluation including audiometry, distortion product otoacoustic emission (DPOAE), electrocochleogram (EcochG), and the vestibular functions, which were evaluated on two scheduled mornings with a battery including vHIT, RCT, and VEMP tests.

Although none of the procedures in this study were experimental, approval was obtained from the Ethics Committee of the Sixth People's Hospital affiliated to the Shanghai Jiao Tong University and was retrospectively registered (Clinical Trial Number: ChiCTR2000029028).

### History-Taking and Questionnaires

The medical history form was adopted from that used by the vestibular unit of our otolaryngology department. It includes questions addressing the audio-vestibular symptoms, the number of vertigo attacks in the past 6 months (26), the duration of vertigo or dizziness, provocation factors, and associated symptoms, among others.

The DHI questionnaire was adopted as reported in a previous study (27). It comprises of 25 questions: seven, nine, and nine questions related to physical (DHI-P), functional (DHI-F), and emotional (DHI-E) aspects of dizziness in MD, respectively. An answer to a question selected as “yes,” “sometimes,” or “no” scored 4, 2, and 0 points, respectively. The participant was asked to focus on the condition in the last 6 months before the recruitment. The total DHI score was counted, and the severity was ranked as mild (0–30 points), moderate (31–60 points), or severe (61–100 points) (28).

### Test Battery of Audio-Vestibular Functions

All tests were performed by qualified medical assistants in a soundproof room. Tympanograms were measured during the first visit of this study using a GSI tympanometer (TympStar; Grason-Stadler, Inc., Denmark) over the pressure range of 200 to −400 daPa at 226 Hz, type A peak was in the range of −100 to +50 daPa, and the static admittance in the range of 0.3–1.6 mho. Otoscopic examinations and tympanometry were completed to ensure normal middle ear functions. Hearing thresholds were determined using a manual audiometer (GSI-61; Grason-Stadler, Inc.) coupled with TDH-39 headphones to obtain PTA threshold across the conventional frequency range of the hearing test (0.5, 1, and 2 kHz). DPOAE was tested using Otometrics OAE system (Madsen Capella^2^, Natus Medical Denmark ApS, Taastrup, Denmark), measured at 2f1–f2 for target frequencies of f2 = 0.5, 1, 2, 4, and 8 kHz, and with f2/f1 set equal to 1.2 and L2 set equal to L1–10. Signal-to-noise ratio equal to or exceeding 6 dB was recorded as normal DPOAE in 0.5–2 kHz. An MEB-9200K evoked potentiometer (NIHON Kohden, Tokyo, Japan) was used to measure EcochGs. The protocol and parameters were reported previously (29). An SP/AP ratio above 0.4 was used as an indicator for the presence of an EH (30).

The vHIT test was employed to address the functions of the three respective SCs on the basis of the selection of the test plane. The standard protocol of Halmagyi et al. (31) was followed in the vHIT test using an ICS Impulse® 3.0 vHIT device (Otometrics A/S; Taastrup, Denmark). The device consists of a lightweight pair of goggles with an integrated video-oculography camera, a mirror to reflect the image of the patient's right eye into the camera, and an inertial system to measure head movements. During the test, participants wearing the goggles were seated with their head and body facing a target dot positioned on a wall at a distance of 1 m. The operator held the subject's head from behind and instructed the subject to look continuously at the dot. The subject's head was quickly turned laterally by 5–15° by the operator at a peak angular velocity of 200–250°/s, as recorded by the system. Responses to unsatisfactory head impulses were excluded. Each participant was subjected to a minimum of 20 head impulses in each of the three planes and to each side (left or right), with unpredictable timing and direction. The ratio between the velocity of the slow-phase eye movement and the velocity of the head impulse was calculated as the VOR HIT gain (23), which was measured in all three planes to evaluate the function of each SC. A HIT gain value lower than 0.8 for the horizontal canal and 0.7 for the anterior and posterior canals with refixation saccades was considered reduced (31–33). A single examiner with specific training in the vHIT procedure who had performed thousands of these tests over more than 3 years was used for this test.

The RCT was utilized to evaluate the function of the H-SC, in response to angular acceleration, at low frequencies from 0.1 to 0.64 Hz at ratio steps (seven frequencies in total). It was performed with a rotatory chair (System 2000; Micromedical, USA) in a completely dark room with the eye movements observed via infrared rays. The participant's head was restrained in 30° forward inclination. After the recording of spontaneous nystagmus, the horizontal gaze-evoked nystagmus and the eye movements in both saccade and pursuit tests were recorded. Finally, the eye movements were recorded using the sinusoidal harmonic acceleration test (SHAT). The eye movements were measured in terms of gain, phase, and symmetry. The result was considered abnormal if the results were out of the normal ranges at two consecutive frequencies (34). Abnormal RCT results suggest a dysfunction in the low-frequency response of the H-SC (35).

VEMPs were used to test the functions of the saccule (inferior vestibular nerve) and utricle (superior vestibular nerve). VEMPs were assessed in a comfortable and quiet environment with a two-channel VEMP System (Neuro-Audio; Neurosoft LTD, Ivanovo, Russia). VEMPs were recorded in response to loud acoustic stimulation to each ear. The acoustical signals consisted of air-conducted 500-Hz tone bursts (2-ms rise/fall, 0-ms plateau) at the level of 100-dB sound pressure level (SPL) delivered by an IP30 insert earphone. The detailed protocol has been described by several previous studies (36–38). The electromyographic (EMG) signal was amplified (5,000 times) and bandpass filtered (30–2,000 Hz). A typical VEMP response consists of an initial positive peak (P1) and a subsequent negative peak (N1). The interpeak (p–p) amplitude (Amp) between P1 and N1 was calculated for both cVEMP and oVEMP that were recorded from the surface electrodes on the sternocleidomastoid muscle and extraocular muscle, respectively. The interaural asymmetry ratio (IAR) was calculated as the ratio between the interaural amplitude difference and the sum. According to the norm data provided by the manufacturer, an absolute value of IAR > 29% and the absence of VEMPs were interpreted as abnormal.

The inner ear is divided into six organs including the three SCs, saccule, utricle, and cochlea. The involvement of each part of the vestibular organ was verified by the abnormality in any of the vestibular test(s) targeting this organ. The cochlea was considered involved in all patient as fluctuating low- to middle-frequency SNHL is one of tough requirements for definite unilateral MD according to the Bárány Society guideline of 2015 (17).

### Statistics

A comprehensive data sheet was used to collect all results including audiometric and vestibular examinations. All data analyses were performed utilizing the IBM SPSS 22.0 (IBM Corp., Armonk, NY, USA) predictive analytics software. Data for categorical and parametric variables are presented in percentages and mean values ± standard deviations (SDs), respectively. The case percentage of an abnormal result in each test was calculated for each group. Fisher's exact test was performed to evaluate the significance of the difference in the test results and DHI across stage groups defined by SNHL. Pearson's correlation was applied to evaluate the relationship between DHI scores and the results of the vestibular tests. An analysis of variance (ANOVA) and Kruskal–Wallis test were carried out to assess data across the stages for normal data and skewed data, respectively. The Mantel–Haenszel chi-squared test was used to assess linear trends between two ordinal categorical variables (39). The correlation analysis of the Spearman rank was used for the correlation analysis of two variables. Next, multivariate linear regression and logistic regression analyses were done to identify the correlation of number of the involved vestibular end organs with DHI. The regression analysis was adjusted for age and sex. Beta coefficient and 95% CI were calculated according to model-variable coefficients and SE, respectively. *P* < 0.05 was considered statistically significant.

## Results

Among the 80 patients, 43 were females (53.75%) and 37 males (46.25%). They were 18–85 years old (mean, 56.7 years). According to the AAO-HNS guideline for PTA-based MD stages (40), participants fell into four groups: 19, 16, 34, and 11 cases in Stage I (≤ 25-dB HL), II (26–40-dB HL), III (41–70-dB HL), and IV (≥71-dB HL), respectively.

In Table 1, the demographic variables, the DHI scores, the frequency of vertigo attacks, and the results of all vestibular function tests are presented for each PTA-stage group. The across-group comparison (by either one-way ANOVA or Kruskal–Wallis test, depending on the nature of the data) showed no significant group effect for any of the variables. The overall null result strongly suggests that PTA-based MD stages are poorly associated with vertigo severity and vestibular functions. There was no significant correlation between DHI scores and SP/AP ratio (*P* = 0.236) in electrocochleogram. No significant differences of DHI scores were found neither between normal and abnormal DPOAE groups (*P* = 0.422) nor between normal and abnormal EcochG (*P* = 0.633).

**Table 1:** Comparison of clinical and test results across different PTA stages.

**PTA stages**	**Total**	**I^a^**	**II^a^**	**III^a^**	**IV^a^**	***P* value**	**Statistic method**
Number	80	19	16	34	11		
Age (years)	56.7 (13.54)	49.73 (14.07)	57.81 (12.22)	58.71 (13.39)	60.91 (12.21)	*P* = 0.071	One-way ANOVA
**Sex**							
Male/female, *n*/*N*	37/43	11/8	8/8	14/20	4/7	*P* = 0.506	Chi-square test
PTA	46.06 (23.80)	14.65 (7.40)	33.54 (3.26)	57.40 (7.83)	83.48 (7.47)	*P* <0.001	One-way ANOVA
DHI total	50.38 (21.20)	48.74 (24.89)	54.12 (20.80)	49.94 (19.86)	49.09 (21.36)	*P* = 0.885	Kruskal–Wallis test
No. of vertigo attacks^*^	4.68 (3.34)	4.89 (3.60)	5.06 (2.84)	3.94 (2.11)	6.0 (5.80)	*P* = 0.668	Kruskal–Wallis test
**Abnormal rates in lab tests, %**							
RCT total	75 (94)	17 (89)	15 (94)	33 (97)	10 (91)	*P* = 0.527	Chi-square test
Gain	38 (48)	9 (47)	8 (50)	15 (44)	6 (55)		
Asymmetry	50 (63)	15 (79)	3 (19)	24 (71)	8 (73)		
Phase	43 (54)	9 (47)	5 (31)	20 (59)	9 (82)		
vHIT total	46 (58)	12 (63)	8 (50)	20 (59)	6 (55)	*P* = 0.880	Chi-square test
H-SC	8 (10)	2 (11)	2 (13)	2 (6)	2 (18)		
A-SC	28 (35)	7 (37)	6 (38)	11 (32)	4 (36)		
P-SC	29 (36)	8 (42)	5 (31)	12 (35)	4 (36)		
VEMP total	53 (66)	13 (68)	10 (63)	25 (74)	5 (45)	*P* = 0.501	Chi-square test
cVEMP	43 (54)	11 (58)	9 (56)	19 (56)	4 (36)	*P* = 0.731	Chi-square test
oVEMP	38 (48)	9 (47)	5 (31)	20 (59)	4 (36)	*P* = 0.275	Chi-square test
No. of involved organs^**^	2.69 (1.20)	2.74 (1.33)	2.56 (1.15)	2.79 (1.15)	2.45 (1.29)	*P* = 0.853	Mantel–Haenszel Chi-square test

*PTA, pure-tone average; DHI, Dizziness Handicap Inventory; RCT, rotatory chair test; vHIT, video head impulse test; H-SC, horizontal semicircular canal; A-SC, anterior semicircular canal; P-SC, posterior semicircular canal; VEMP, vestibular-evoked myogenic potential; cVEMP, cervical vestibular-evoked myogenic potential; oVEMP, ocular vestibular-evoked myogenic potential*.

* *Vertigo attacks in the last 6 months*.

** *Number of involved vestibular end organs*.

a *Continuous data are presented as mean (SD) values; otherwise, data are reported as number (percentage)*.

In this sample, the disease duration since the onset of MD ranged from 6 months to 50 years. Correspondingly, the total DHI scores and the frequency of the vertigo attacks also largely varied across individuals. The total DHI score was on average 50.38 (SD, 21.20), ranging from 0 to 92 with 50 as the median [interquartile range (IQR), 38.5–63.5]; the mean number of vertigo attacks was 4.68 times (SD, 3.34), ranging from 0 to 21 times with 4 as the median (IQR, 3–6) in the 6-month period before this study.

**Figure 2:**
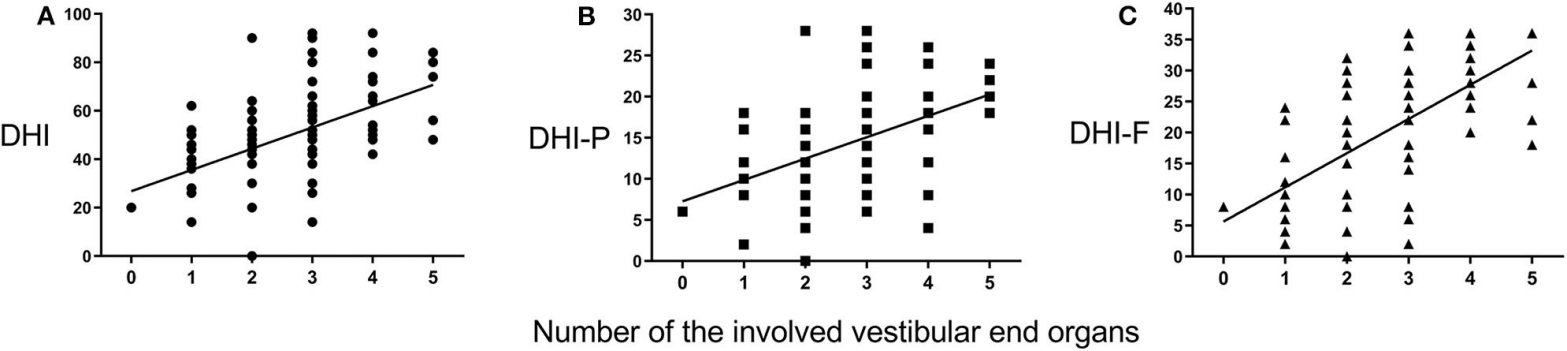
Correlations between Dizziness Handicap Inventory (DHI) scores and number of involved vestibular end organs. The DHI **(A)**, DHI-P **(B)**, and DHI-F **(C)** scores positively correlate with the number of involved vestibular end organs.

**Figure 3:**
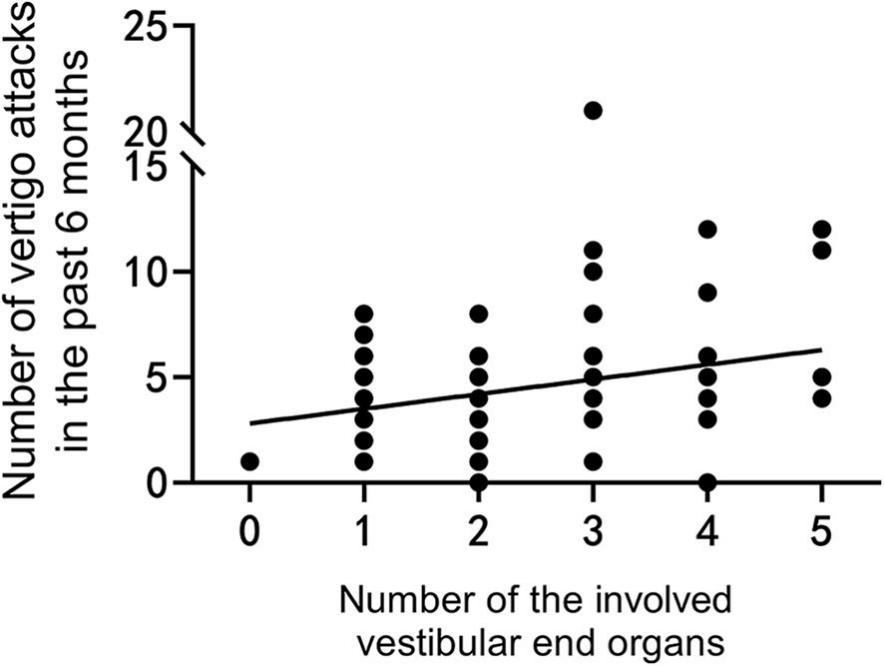
The number of vertigo attacks is positively correlated with the number of involved vestibular end organs.

The number of the involved vestibular end organs from 0 to 5 with 3 as the median (IQR, 2–3.75), and 2.69 was the average in this sample. The number of the involved vestibular end organs was not significantly correlated with the PTA stages according to the Mantel–Haenszel chi-squared test (χ^2^ = 0.052, *P* = 0.853). However, the number of the involved vestibular end organs was positively associated with the total DHI score (*P* < 0.001, *r* = 0.521; Figure 2A), as well as the scores for DHI-P and DHI-F (*r* = 0.506, *P* < 0.001, Figure 2B; *r* = 0.556, *P* < 0.001, Figure 2C; respectively). The number of the involved vestibular end organs was also positively associated with the number of vertigo attacks in the past 6 months (*r* = 0.238, *P* = 0.034; Figure 3). Furthermore, the DHI score was positively associated with the frequency of vertigo attacks (*r* = 0.243, *P* = 0.030 for the total DHI score; and *r* = 0.236, *P* = 0.035; *r* = 0.271, *P* = 0.015; and *P* > 0.05 for DHI-P, DHI-E, and DHI-F, respectively).

**Figure 4:**
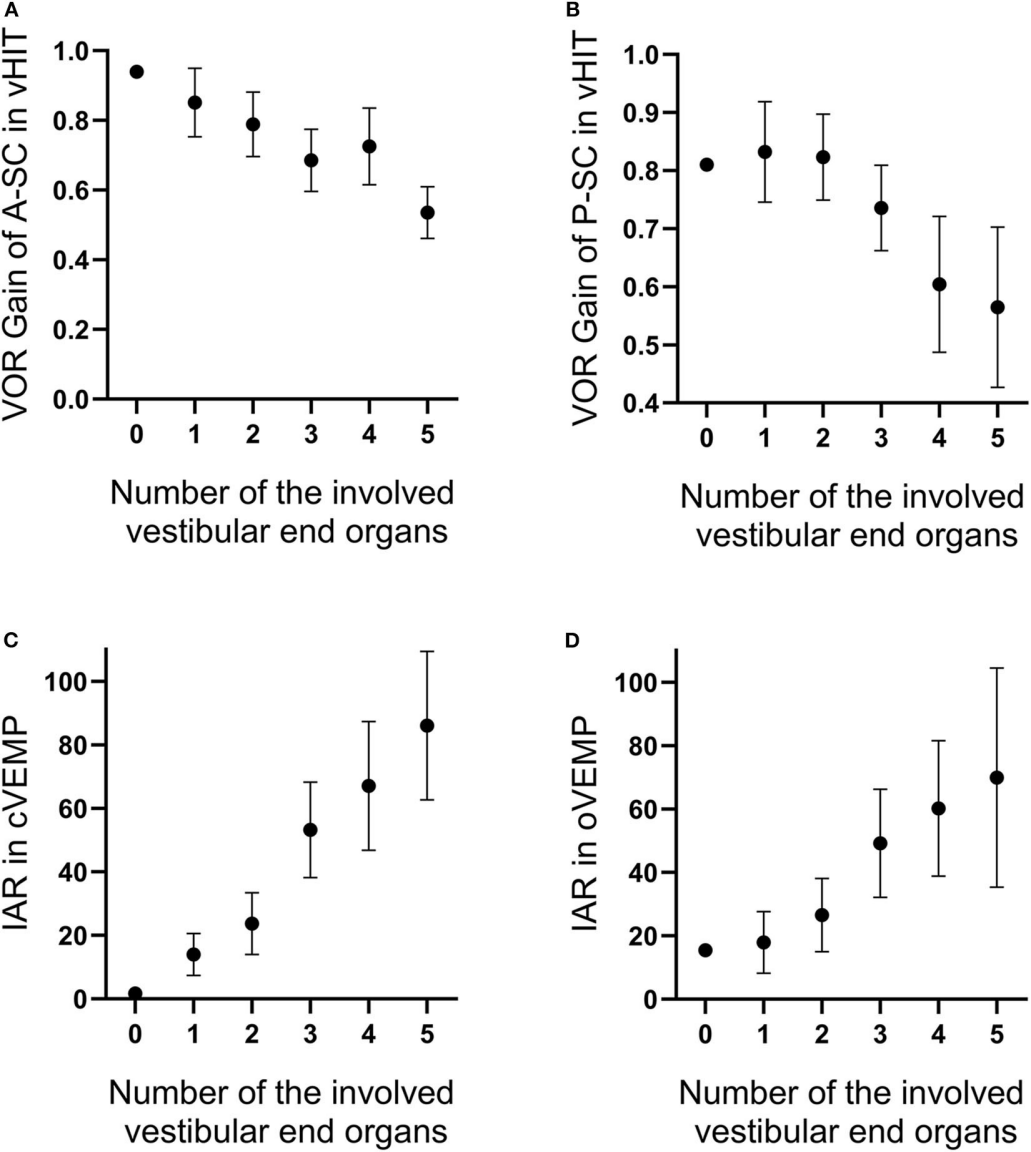
The number of involved vestibular end organs is negatively correlated with the vestibulo-ocular reflex (VOR) gains of anterior **(A)** and posterior **(B)** semicircle canals tested in video head impulse test (vHIT) and is positively correlated with interaural asymmetry ratios in vestibular-evoked myogenic potentials (VEMPs) **(C,D)**. The range of error bars is the expression of 95% confidence interval.

We also found that the number of the involved vestibular end organs was significantly correlated with several vestibular function parameters obtained in tests. The number of the involved vestibular end organs was negatively associated with VOR gains of A-SC (*P* = 0.001, *r* = −0.402; Figure 4A) and P-SC (*P* < 0.001, *r* = −0.486; Figure 4B) in vHIT and was positively associated with IAR in cVEMP (*P* < 0.001, *r* = 0.679; Figure 4C) and oVEMP (*P* < 0.001, *r* = 0.499; Figure 4D).

**Figure 5:**
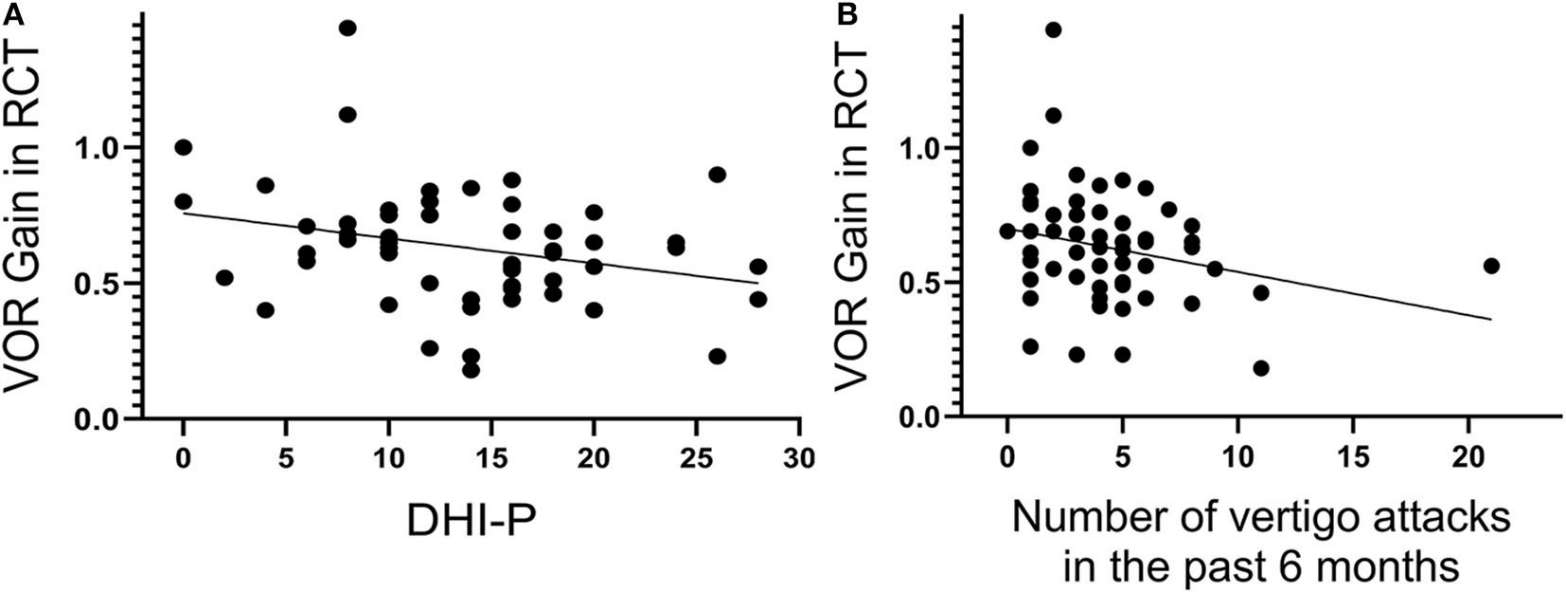
The vestibulo-ocular reflex (VOR) gain of the rotatory chair test (RCT) at 0.08 Hz significantly correlates with the Dizziness Handicap Inventory–physical (DHI-P) score **(A)** and number of vertigo attacks in the past 6 months **(B)**.

Moreover, the VOR gain obtained in the RCT was significantly correlated with the number of vertigo attacks in the past 6 months (*P* = 0.047, *r* = −0.264; Figure 5A) and the DHI-P scores (*P* = 0.032, *r* = −0.285; Figure 5B) at 0.08 Hz. This positive correlation suggests that the VOR gain in the RCT may quantitatively reflect the degree of an H-SC pathology. On the other hand, the DHI scores were positively correlated with IAR in cVEMP (*P* = 0.001, *r* = 0.366 for the total DHI score; and *r* = 0.377, *P* = 0.001; *P* > 0.05; and *r* = 0.456, *P* < 0.001 for DHI-P, DHI-E, and DHI-F, respectively) and oVEMP (*P* = 0.004, *r* = 0.322 for the total DHI score; and *r* = 0.235, *P* = 0.036; *P* > 0.05; and *r* = 0.332, *P* = 0.003 for DHI-P, DHI-E, and DHI-F, respectively). However, no such significant correlation was observed between the DHI scores and the results of the vHIT (all *P* > 0.05).

We considered sex, age, the number of the involved vestibular end organs, frequency of the vertigo attacks, PTA, VOR gains of three SCs, and IAR in VEMPs as potential factors with DHI. In our study, age and sex were adjusted in the model. In the multivariate linear regression analysis, total DHI score was positively correlated with the number of the involved vestibular end organs (beta coefficient, 10.653; 95% CI, 5.031–16.275; *P* < 0.001). In the logistic regression analysis, mild and moderate DHI ranks were combined to mild/moderate DHI; we established a model in that the number of the involved vestibular end organs could predict severe DHI statistically significantly (odds ratio, 4.724; 95% CI, 1.597–13.974; *P* = 0.005); the model could predict 80% of the subject in correct class. The variables found to be significant predictors in the model were the number of the involved vestibular end organs (odds ratio 4.724) and PTA (odds ratio 0.966, *P* = 0.037).

Among all vestibular tests, the abnormal rate was the highest in the RCT (93.75%), followed by cVEMP and oVEMP (53.75 and 47.5%, respectively), vHIT for the P-SC (36.25%), A-SC (35%), and H-SC (10%). The abnormal rate of the H-SC was significantly higher in the RCT than in the vHIT test for the same SC (93.75 vs. 10%, chi-squared test). The H-SC appears to have a much lower abnormal rate than the other two SCs, suggesting a lower incidence of its involvement in MD.

If the abnormal rates for SCs are cumulatively counted for the membranous SCs (i.e., the SCs are considered abnormal if any of the SC tests is abnormal) and if the same principle is applied to the combined abnormal rates for the vestibular part of the membranous labyrinth (saccule and utricle) evaluated by oVEMP and cVEMP tests, then our data show an abnormal rate of 57.5% for the membranous SCs, which is slightly lower than the accumulated rate of 66.25% for the vestibular parts (*P* = 0.329, chi-squared test).

**Figure 6:**
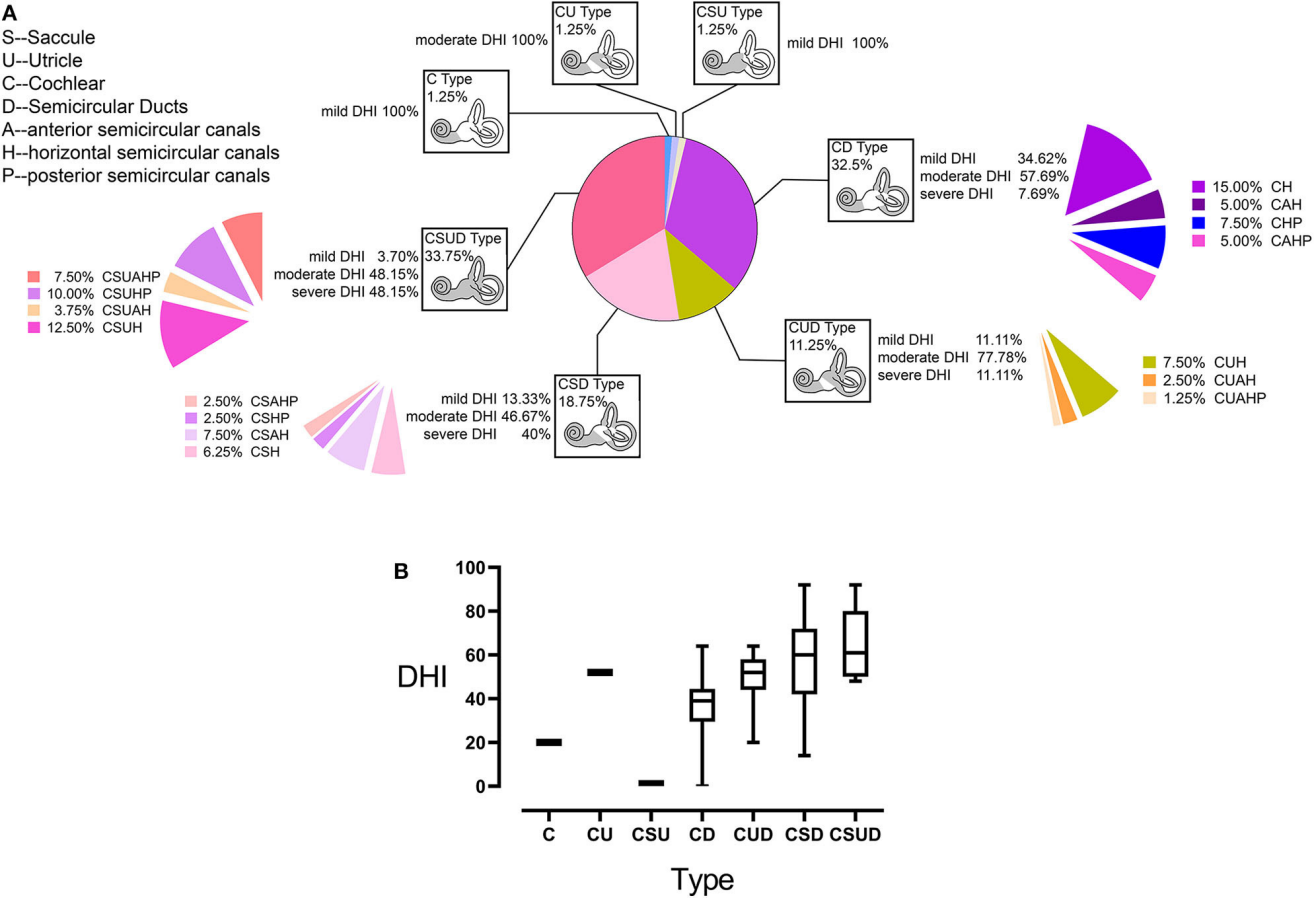
Categorization of study participants with Menière's disease (MD) based on the involved parts of the inner ear **(A)**. The involvement of the cochlea was labeled as the C (cochlea) type pathology in all subjects. Similarly, the involvement of the saccule, utricle, and semicircular canals (SCs) was labeled as the S-, U-, or D-type pathology, respectively. In the D type, the involvement of a specific SC was labeled by the suffix H, P, and A for H-SC, P-SC, and A-SC, respectively. The type and percentage of participants for each type out of the total study population are presented inside each box. The percentages of participants with each Dizziness Handicap Inventory (DHI) level relative to the total population of this type are listed beside each box. Moreover, the patients with the involvement of different SCs are listed as percentages out of the total sample. Except for three types with only one patient, DHI score and the number of the involved vestibular end organs are increased clockwise **(B)**; the range of error bars is the expression of 95% confidence interval.

We use the abnormality of a test to represent the abnormality of the organ targeted by the test, when categorizing all participants into seven groups on the basis of the involvement of different organs in the inner ear (Figure 6A). The seven groups are defined by individual or combined type(s) of pathologies. Only a few individuals were categorized as C, CU, or CSU types. As shown in Figure 6B, except the three types with only one patient, DHI score and the number of involved vestibular end organs are increased clockwise.

## Discussion

In the present study, we found that neither the clinical symptoms nor the abnormal rates of each individual vestibular test were significantly different across the four MD stages (Table 1) that were defined by the degree of HL. The null results suggest that the HL-based four-stage system does not appropriately represent the severity of MD. On the other hand, the number of the involved vestibular end organs appears to be a good, objective indicator of disease severity, because it is significantly correlated with the vertigo severity indicated by the clinical evidence (both the DHI score and the frequency of the vertigo attack). Moreover, the number of involved vestibular end organs was significantly associated with several vestibular function parameters such as IAR in cVEMPs and oVEMPs, and VOR gains of posterior/anterior canal in vHIT. The model used to separate severe dizziness handicap from mild/moderate dizziness handicap was able to explain 80% of the variance in the logistic regression analyses; the number of involved vestibular end organs can increase the odds of achieving severe DHI. The predictive model was reasonable and needs to be verified in a prospective study. A battery of vestibular tests targeting different vestibular end organs is a complementary method for evaluating inner ear deficits and aid in the “grading” of potential disease with MD.

A large number of previous studies have investigated the changes of individual vestibular tests across different stages of MD. For example, VOR gain in impulse rotatory tests was concluded to be affected by the stage (41), which is higher during attacks in the early stages but decreased in the later stages. This was consistent with finding in another study that showed normalized or even increased VOR gains in patients with stage 1 and 2 MD after cessation of attacks, whereas abnormality in patients with stage 3 MD remained (42). The correlation between IAR and MD stages was suggested by in VEMP test in one study (19). Moreover, cVEMP absence was highly correlated with hearing loss in the other study (43). Further, VEMPs were used to indicate control of vertigo caused by MD (44), and cVEMP was suggested to be implemented to track progression of MD (45). These studies suggested that the test results of vestibular functions might be helpful to identify the progress of MD. However, different opinions were recently presented. For example, Van Esch found that abnormal vHIT results was seen in the later stage of MD, but there was no clear relationship between the incidence of abnormal vHIT and the stages of disease (46); Fukushima et al. concluded that vHIT was not dependent on volume of EH assessed in MRI (47); and Rubin found that vHIT was normal between vertigo attacks in the advance of unilateral definite MD between vertigo attacks (48). Furthermore, a few studies showed an overlap in VEMP values between MD patients and normal subjects (49, 50). In each of the studied cited above, it is not surprising that a test targeting a single organ is unable to assess MD progress independently, but all parts are complementary to each other when used to evaluate MD. Montes-Jovellar was the first who used a comprehensive approach in tracking the progress of MD. His study identified four groups of MD patients according to auditory, vestibular, posturographic, and disability assessments (51).

Based on the technologies that are currently available, the results of this study suggest that the number of involved vestibular end organs is a reliable indicator of MD severity. The number is well correlated with the severity of clinical symptoms including DHI score and the number of vertigo attacks (Figures 2, 3). We propose this index as a temporary supplement for the current four-stage system that is purely based on HL. This staging system should be refined because it limitedly represents the status of disease progression in patients with MD (52, 53).

Among all vestibular tests, the abnormal rate was the highest in the RCT, which evaluates H-SC responses to low-frequency rotation. This was followed by the cVEMP and oVEMP tests that target the saccule and utricle, respectively, and finally by the vHIT, which assesses high-frequency responses of the P-SC, A-SC, and H-SC. The H-SC involvement appears to be the most frequent as it is seen in almost all subtypes with a D-type pathology (Figure 6A). This most frequent involvement is likely due to the highest abnormal rate in the RCT but does not really indicate a higher chance of an H-SC pathology as indicated by the difference in the abnormal rates (93.75% in the RCT for H-SC and <40% in the vHIT for A- and P-SCs). The results of the RCT and vHIT are often dissociated in MD, so as caloric test and vHIT, reported by other studies (54–56). Caloric test was represented more sensitive than the vHIT test previously, whereas the caloric test was abnormal only when vestibular dysfunction in RCT was detected at least three consecutive frequencies (34). However, the vHIT performed better specificity in detecting abnormal SC function (57).

An accumulation of endolymph in the cochlea and the vestibular system in the inner ear was often observed as a characteristic feature associated with MD (12). However, the relationship between EH and frequency of vertiginous attacks is still unclear. Caloric asymmetry was found dependent on the EH volume, but vHIT does not (47). Fukushima et al. proposed that canal paresis on caloric test was caused by small cupula amplitude when the volume of membranous labyrinth is large, whereas a large cupula amplitude generated by high-frequency angular acceleration in vHIT was not affected by the volume. However, hydrodynamic theory might not be the appropriate explanation for the dissociation of RCT and vHIT. Gates thought that MD may be a channelopathy (58) and that EH will modify sodium homeostasis in separation between endolymph and perilymph (59, 60); for example, ion transport function of the endolymphatic sac epithelium was considered to be critically involved in the etiology of MD symptoms. Such hypotheses remain to be further studied and confirmed by direct evidence.

The dissociation may be due in part to different characteristics of the stimuli applied for each test based on the pathophysiology of type I and II hair cells, as well as different afferent fiber types. Given the fluctuating clinical presentation of MD, the differing results can be ascribed to the predilection for type II hair cell loss in MD (54, 61). It has been suggested that type I hair cells and irregular afferent fibers are responsible for the high-frequency response in the vHIT, whereas the type II hair cells and regular fibers are responsible for the low-frequency (caloric) response (56). Rubin demonstrated the phasic hair cells with irregular fibers were still functioning in advanced stage of MD, which contributed to normal vHIT results in contrast to abnormal low-frequency reflex (48). During the attacks of MD, varied results of vHIT and usually decreased caloric responses suggested a frequency-dependent impairment in MD (42). In a word, vestibular tests addressing different frequencies are complementary to each other for vestibular function assessments in MD.

On the other hand, differences in abnormality rates among these different tests cannot tell the susceptibility of different organs that are targeted by those tests, because the sensitivity and specificity vary across different tests. For example, the highest abnormality in the RCT (targeting H-SC) does not suggest a much higher susceptibility of the H-SC in comparison with the two other SCs, at least not as big as the differences in the abnormal rates between the RCT for H-SC and the vHIT for the other two SCs suggest. Currently, the vHIT is the only test that can evaluate multiple organs. In this test, the abnormal rate was the lowest in the H-SC plane, suggesting that the H-SC is less likely to be involved in MD progression. SC dysfunction in MD was reported most frequently locating in the P-SC (47, 62), and VOR gain reduction of S-HC occurred more frequently than H-SC (62, 63). In our study, vertical vHIT were indispensable despite lack of low-frequency function assessment in vertical SCs.

In the present study, on the basis of a vestibular test battery comprising RCT, vHIT, cVEMP, and oVEMP that has recently been utilized for mapping inner ear pathology in MD, we counted the number of involved vestibular end organs, which was significantly associated with IARs in VEMPs and VOR gains of vertical semicircle canals in vHIT. These parameters were either related to risk of balance disorder and subsequent injuries (64) or be correlated to subjective sensation of MD symptoms (47). We intended to categorize the types of MD on the basis of the involvement of different inner ear organs. Although the number of the involved organs is not quantitative *per se*, owing to limitations of the current technology, it provides interesting insights for further investigations and may have significant implications in clinical practice.

Firstly, the categorization of MD based on the organ involvement may help understand the development of MD better. Based on our data, the cochlea was most frequently involved in the present study population, and cochlear abnormalities were detected in participants with mild DHI. This high prevalence of cochlear involvement was followed by those of the saccule, utricle, and SCs. We conclude that MD is most likely to originate in the cochlea and spread to the saccule, utricle, and SCs discontinuously (11). Furthermore, simple SC involvement was mainly detected in patients with mild and moderate DHI, whereas diffuse involvement (CSUD type) was observed in the largest number of patients with moderate and severe DHI (33.75%, Figure 6); the separate or combined involvement of the saccule and utricle was detected with higher DHI scores than simple SC involvement, which indicated the damage of the saccule and utricle contribute to more disability experienced by patients with MD.

Secondly, the organ-type categorization may provide further insight into clinical management. For example, steroids can reduce the frequency of vertigo attacks (65, 66), based on their potential in controlling the EH. Steroid treatment through intratympanic perfusion in animals suggests a higher endolymphatic concentration than do systemic routes (67), and methylprednisolone in humans also produces high perilymph concentrations (68). Such treatment may be more effective in patients with C and S types than the D type because of the longitudinal flow theory in endolymphatic circulation and absorption, which describes that endolymph fluid is partly derived from filtration of the perilymph through Reissner's membrane.

The study is not population-based, and this bias is a big limitation for using this system as an ideal staging method. Far from being the final word on the topic, we offer this classification as a starting point for future discussions, and we are looking forward to more population-based studies. On the other hand, there several other methodological limitations. Firstly, DHI was evaluated for the study group only in one occasion, which might cause bias because of faint memory or subjective feeling. Secondly, recent studies confirmed that there is an overall shift to a higher resonant frequency in ears with MD, but only a single study frequency of 500 Hz was considered in present study, which might not reflect an altered biomechanical system in MD correctly.

Although vestibular function measures are superior to the staging system based solely upon the degree of HL, using a single number of involved vestibular end organs as the indicator of MD severity is coarse and limited by three weaknesses: lack of VOR assessments of vertical SCs in low frequency, currently the functional deficits of the involved organ cannot be quantified, and the sensitivity of the vestibular tests is generally unknown or low. Although each individual vestibular test returns a quantitative value, currently, each test cannot determine the degree of functional loss for targeted organ. On the other hand, overall, the sensitivity and specificity of all vestibular tests are not at a satisfactory level, so the test results may not accurately represent the degree of vestibular pathologies (57, 69–76).

## Conclusions

Our results suggest that the MD stages based on the degree of hearing loss do not reflect the severity of the disease. The number of involved vestibular end organs is a useful and objective indicator of MD severity. A battery of vestibular tests targeting different vestibular end organs is a complementary method for evaluating the inner ear deficits and aid in “grading” of potential disease with MD. However, currently, the damage of vestibular end organs cannot be quantified sufficiently. Further improvements and investigations are needed to increase the accuracy of this test battery.

## Data Availability Statement

All datasets generated for this study are included in the article/Supplementary Material.

## Ethics Statement

The studies involving human participants were reviewed and approved by Ethics Committee of the Sixth People's Hospital affiliated to the Shanghai Jiao Tong University. The patients/participants provided their written informed consent to participate in this study.

## Author Contributions

SH, HW, and SY designed and coordinated the study. SH analyzed the data and wrote the manuscript. YF, DY, and HS supervised the data collection. SH, HZ, and EZ performed the data collection. SH, HW, JW, and SY interpreted findings and reviewed the manuscript. All authors contributed to the article and approved the submitted version.

## Conflict of Interest

The authors declare that the research was conducted in the absence of any commercial or financial relationships that could be construed as a potential conflict of interest.
